# Target identification strategies in plant chemical biology

**DOI:** 10.3389/fpls.2014.00352

**Published:** 2014-07-24

**Authors:** Wim Dejonghe, Eugenia Russinova

**Affiliations:** ^1^Department of Plant Systems Biology, VIBGhent, Belgium; ^2^Department of Plant Biotechnology and Bioinformatics, Ghent UniversityGhent, Belgium

**Keywords:** small molecule, target identification, plant biology, chemical genetics, *Arabidopsis thaliana*

## Abstract

The current needs to understand gene function in plant biology increasingly require more dynamic and conditional approaches opposed to classic genetic strategies. Gene redundancy and lethality can substantially complicate research, which might be solved by applying a chemical genetics approach. Now understood as the study of small molecules and their effect on biological systems with subsequent target identification, chemical genetics is a fast developing field with a strong history in pharmaceutical research and drug discovery. In plant biology however, chemical genetics is still largely in the starting blocks, with most studies relying on forward genetics and phenotypic analysis for target identification, whereas studies including direct target identification are limited. Here, we provide an overview of recent advances in chemical genetics in plant biology with a focus on target identification. Furthermore, we discuss different strategies for direct target identification and the possibilities and challenges for plant biology.

## Introduction

A proven way to study how something works is to perturb the process of interest in a well-defined and controlled manner. In biology, this is often accomplished by introducing alterations into the genome of an organism, such as mutations or ectopic expression. A major disadvantage of working at the gene level is that the resulting organism will live in a steady state with the induced genetic change. Additionally, perturbations of essential gene functions will lead to lethality, unless conditional, and perturbations of a gene member of a large gene family might have no effect due to redundancy. In order to address gene redundancy and lethality problems, together with the possibility to perturb a system in a more dynamic manner, chemical biology approaches can be used. In chemical biology, typically small molecules are applied to a biological system, altering the process of interest by binding target molecules. A key feature of chemical biology is its conditional nature. Small molecules can be used for any desired time and concentration, and in most cases can be washed out of the system of choice, making them an ideal tool to study dynamic processes for a certain period of time. Crucially, small molecules will not alter an organism over generations and are not restricted to bind only proteins, but can modulate a biological system by binding lipids or nucleic acids (Ziegler et al., 2013). Finally, using different approaches, the target of small molecules needs to be identified to get a better understanding of the affected process.

Chemical genetics strategies in plant biology lag behind the animal field, in which drug development provided a plethora of different target identification strategies. In plant biology, most target identification strategies consist of a phenotyping approach or a forward genetics strategy based on small molecule resistance screens. A few examples exist of strategies, such as affinity purification, that were successfully applied (Tresch, 2013).

**Figure 1:**
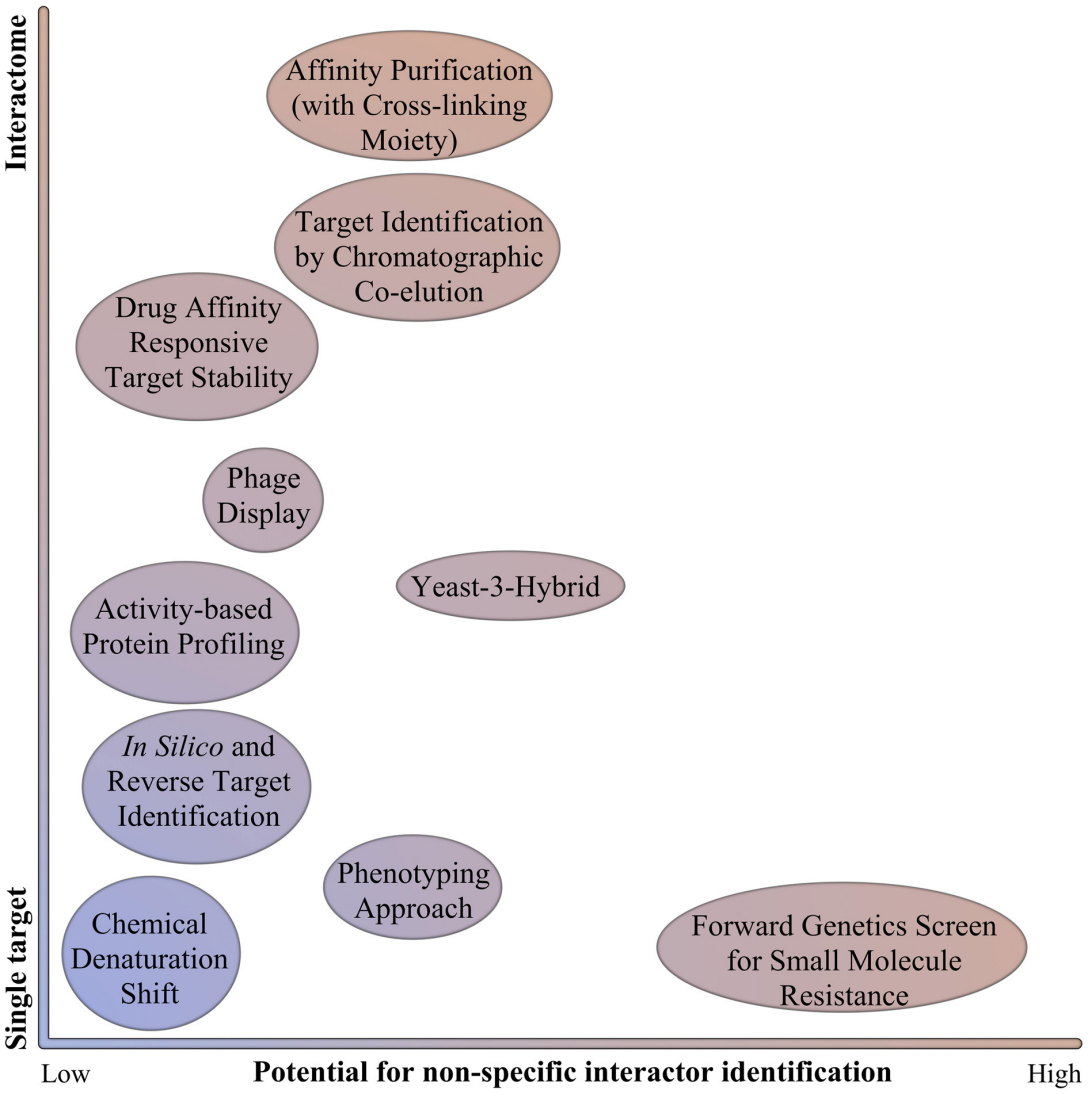
**Schematic representation of target identification strategies**. Target identification strategies are represented in function of their ability to identify only one target or several targets (interactome), and the potential to identify non-specific interactors (such as proteins that will confer resistance or induce the appropriate readout without actually binding specifically the small molecule).

An important aspect of chemical biology is linking the induced phenotype to one or more targets (Figure 1). Usually, only the relevant target, or target with the highest affinity for the small molecule, is identified and validated, although so-called “off-targets” might contribute substantially to the overall phenotype. Therefore, it has become increasingly important to understand and generate the small molecule interactome, in order to explain the observed phenotypes (Lounkine et al., 2012). This aspect is especially important for small molecules with a commercial application in healthcare or agriculture.

Several reviews have addressed the chemical genetics approaches in plant biology and the challenges and opportunities that lay ahead (Tóth and van der Hoorn, 2010; Kumari and van der Hoorn, 2011; Hicks and Raikhel, 2012; Xuan et al., 2013). Other recent reviews include a comprehensive overview of target identification strategies in mostly animal systems (Ziegler et al., 2013), and a thorough overview of small molecules with known targets and mode of action in plant biology (Tresch, 2013). Given that plant biological research uses limited target identification approaches, this review will briefly discuss the current ones, and will mostly focus on emerging new strategies, which have not found a broad application in plant biology yet (Table 1). Where applicable, examples from plant biology will be given, and benefits and shortcomings will be discussed. The ultimate aim of this review is to convince the reader to look further than the established target identification strategies when a chemical genetics approach is considered.

**Table 1:** **Overview of the target identification strategies**.

**Target identification strategy**	**Examples^a^**	**Modified small molecule**	**References**
**ESTABLISHED STRATEGIES**			
Forward genetics screen for small molecule resistance	Pyrabactin, gravacin, DAS734	No	Rojas-Pierce et al., 2007; Walsh et al., 2007; Park et al., 2009
Phenotyping approach	Bikinin, kynurenine, imprimatins	No	De Rybel et al., 2009; He et al., 2011; Noutoshi et al., 2012
*In silico* and reverse target identification	IGPD inhibitors, galvestine1 and galvestine2	No	Schweitzer et al., 2002; Botté et al., 2011
**EMERGING STRATEGIES**			
Activity-based protein profiling	Bicyclic hydantoin, serine hydrolases	Yes	Kaschani et al., 2012a,b
Yeast-3-Hybrid	Jasmonic acid, abscisic acid, compound 8, cucurbic acid, cucurbic acid methylester, 2,6 dihydroxybenzoic acid	Yes	Cottier et al., 2011
Affinity purification with cross linking moiety	Atrazine, jasmonate glucosate, castasterone	Yes	Pfister et al., 1981; Kinoshita et al., 2005; Nakamura et al., 2008
Phage display	Brz2001	Yes	Takakusagi et al., 2013
**PROMISING STRATEGIES**			
Affinity purification	None yet	Yes	Ziegler et al., 2013
Chemical denaturation shift	None yet	No	Schön et al., 2013
Target identification by chromatographic co-elution	None yet	No	Chan et al., 2012
Drug affinity responsive target stability	None yet	No	Lomenick et al., 2009

a *The examples correspond with those given in the text*.

## Commonly used target identification strategies in plant chemical biology

### Forward genetic screen for compound resistance

Forward genetic screen for compound resistance is a commonly used target identification strategy in plant chemical biology in which a mutant population is grown in the presence of small molecules and screened for resistance. Selected resistant individuals are subsequently characterized in terms of their mutations. A major disadvantage is the inherent selection against targets with an essential gene product, provided that the induced mutation causes a knock-out or renders the protein inactive. Essential gene targets might still be selected when the mutation allows proper protein function, but inhibits the small molecule from binding. Additionally, gene redundancy can prevent identification of the target, and certain mutations might make plant resistance to compound treatment, without affecting the true target. A small molecule called “non-auxin-like lateral root inducer” or naxillin illustrates the latter scenario. Identified from a screen for small molecules able to enhance lateral root development, naxillin was found to affect lateral root development more specifically than auxin. The only identified resistant mutant from an ethylmethane sulfonate (EMS)-mutagenized *Arabidopsis* population, *naxillin resistant 1* (*nar1*), proved to be affected in the *INDOLE-3-BUTYRIC ACID RESPONSE 3* (*IBR3*) gene, which is involved in the conversion of indole-3-butyric acid (IBA) to indole-3-acetic acid (IAA). The characterization of *nar1* helped to reveal that naxillin acts upstream of auxin signaling by positively affecting the IBA to IAA conversion at specific sites in the root, thereby inducing lateral root development, but failed to identify the true target of naxillin (De Rybel et al., 2012). Major advantages of the forward genetic screen approach are the straightforward experimental setup and the availability of high-throughput next-generation sequencing techniques, which allow relatively quick target identification once the resistant individuals are isolated.

Whether or not mutants are more sensitive to the compound depends on the nature of the mutation. Resistant mutants might arise from mutations affecting the small molecule binding, or from the absence of the target protein, although the latter is not applicable to proteins with essential function. Alternatively, less of the protein target might result in hypersensitivity, as less small molecule is required to exert the same phenotypic effect. Besides, resistance to small molecules might also be caused by the overexpression of the target protein.

Examples of such an approach are the identification of targets for pyrabactin, gravacin and DAS734. The synthetic seed germination inhibitor pyrabactin was shown to act as a specific agonist of abscisic acid (ABA) because transcriptional responses of seeds growth in presence of ABA compared to pyrabactin were highly correlated, whereas this was not the case in seedlings (Park et al., 2009). Pyrabactin allowed the identification of the PYR/PYLs (for “pyrabactin resistance” and “PYR-like”), members of the ligand binding cyclase subfamily of the START protein superfamily, through a forward genetics screen for compound resistance (Park et al., 2009). This protein family was independently identified as RCAR (for “regulatory component of ABA receptor”) (Ma et al., 2009). The PYR/PYL/RCARs were shown to be ABA receptors (Park et al., 2009), which after perception bind to type 2C protein phosphatases, thereby inactivating them. The role as ABA receptor for the PYR/PYL/RCAR protein family was later confirmed by crystallographic data (Santiago et al., 2009).

Gravacin was identified as an inhibitor of the gravitropic response in *Arabidopsis* seedlings (Surpin et al., 2005). A population of 220,000 EMS-mutagenized F2 seeds were screened for a gravitropic response when grown on gravacin, identifying through a map-based cloning approach an E to K substitution in the gene coding for P-GLYCOPROTEIN 19 (PGP19) (Rojas-Pierce et al., 2007). Several different mutant alleles for PGP19 showed resistance to gravacin, confirming the identified mutation as the cause of gravacin resistance. Furthermore, gravacin binding to PGP19-containing microsomes was severely reduced in *pgp19* mutants compared to wild type controls (Rojas-Pierce et al., 2007).

A phenyltriazole acetic acid compound, DAS734, was identified as a potent bleaching agent of developing leaves. Addition of adenine could alleviate the effects, hinting toward a target in the purine biosynthesis pathway (Walsh et al., 2007). A screen for DAS734 resistance of 480,000 EMS-mutagenized *Arabidopsis* ecotype Col-0 seedlings resulted in several resistant lines, some of which had the same mutation. Map-based cloning identified *GLUTAMINE PHOSPHORIBOSYLAMIDOTRANSFERASE 2* (*GPRAT2*) as the gene containing all mutations (Walsh et al., 2007). Expression of *AtGPRAT2* in *Escherichia coli* allowed the purification of the protein and the evaluation of its activity in the presence of DAS734. The small molecule was able to potently inhibit GPRAT2 activity in a slow, but reversible manner. In addition, expression of the mutant *GPRAT2* gene in *E. coli*, isolated in the forward genetics screen, revealed an increase in the inhibitory concentration (IC_50_) of more than 500 times, indicating a strong resistance to DAS734, and confirming GPRAT2 as its target (Walsh et al., 2007).

### Phenotyping approach

Opposed to a forward genetics approach, the phenotyping approach usually starts from a screen of small molecules against an appropriate readout for the biological process of interest, followed by further tests that narrow down the possible target proteins. Biochemical validation is used to confirm the hypothetical target protein. The main drawback is the requirement for proper readouts that is applicable for known signaling pathways and enzymes involved in primary and secondary metabolism. Processes of highly organized, rapid and dynamic nature, such as endomembrane trafficking, will be much harder to characterize with such a strategy. As one searches specifically within a process of interest, this approach usually will yield only one target, or target family, but will not provide an overall picture of small molecule interactors. The latter implies, however, that target identification can be fairly straightforward because the search is directed. Some recent examples are the identification of targets for bikinin, kynurenine and imprimatins.

The small molecule bikinin was discovered in a screen for molecules able to induce phenotypes similar to those caused by the application of the most active brassinosteroid (BR), brassinolide, in young *Arabidopsis* seedlings (De Rybel et al., 2009). The target of bikinin was identified through comparative phenotypic analysis of different BR-related mutants grown on bikinin. As bikinin was able to rescue a gain-of-function *bin2-1* mutant to wild type, it was hypothesized that the GSK3-like kinase BIN2 is the direct target. This hypothesis was confirmed by *in vitro* binding studies. In addition, the list of bikinin targets was expanded to other *BIN2* homologs and a competition with ATP was suggested as the mode of compound action.

The selective aminotransferase inhibitor, affecting local auxin biosynthesis, l-kynurenine (Kyn), was identified in a screen for suppressors of the constitutive ethylene response (He et al., 2011). Although Kyn did not rescue the constitutive ethylene response phenotypes of the *eto1-2* and *ctr1-1* mutants and wild type plants treated with the synthetic ethylene precursor 1-aminocyclopropane-1-carboxylic acid, it rescued the shortened root phenotype at submicromolar concentrations. Kyn was shown to inhibit ETHYLENE INSENSITIVE 3 (EIN3) accumulation in *Arabidopsis* roots, which led to a reduction in local auxin responses. As active ethylene signaling increased the reduction of local auxin responses in the presence of Kyn, it was concluded that Kyn represses ethylene-mediated auxin responses (He et al., 2011). Further unraveling of auxin responses led to the hypothesis that Kyn might inhibit TRYPTOPHAN AMINOTRANSFERASE OF ARABIDOPSIS1 (TAA1). Enzymatic activity tests on purified TAA1 confirmed that Kyn is a competitive and potent inhibitor, inhibiting the conversion of tryptophan to indole-3-pyruvic acid. Computational modeling validated Kyn as a competitive inhibitor of TAA1, outcompeting tryptophan. In addition, when tryptophan was applied in excess, it reversed the inhibitory effects of Kyn (He et al., 2011).

The third example comes from a screen for small molecules affecting disease resistance in plants that identified five small molecules belonging to two different structural groups, named imprimatins (Noutoshi et al., 2012). The authors showed an increase in salicylic acid (SA) in treated plants, but unlike control plants, after pathogen infection imprimatins did not accumulate the inactive form of SA, SA-2-O-β-D-glucoside (SAG), which usually increases in parallel with an increase in SA. An enzymatic test on UGT74F1 and UGT76B1, two enzymes that convert SA to its inactive form SAG, confirmed imprimatins as inhibitors of these enzymes. Thus, the increased *Pst-avrRpm1-induced* cell death after imprimatin treatment is due to an inhibition of the SA-to-SAG conversion (Noutoshi et al., 2012).

### *In silico*-based target identification strategies

Not only forward, but also reverse and *in silico* design strategies have been successfully used. The starting point is a protein of interest, or a small molecule scaffold. Screening of additional small molecules aims at finding a specific interactor for the protein of interest, or at improving binding characteristics for an existing small molecule. A validation step *in vivo* confirms the findings of the reverse or *in silico* strategy.

A first example concerns a study of more than a decade ago in search of novel inhibitors of imidazole glycerol phosphate dehydratase (IGPD), an attractive herbicide target (Schweitzer et al., 2002). Based on previously identified IGPD triazole inhibitors (Mori et al., 1995), a pharmacophore model was developed to search available 3D-databases (Schweitzer et al., 2002). A pharmacophore model contains spatial information on functional groups essential for small molecule action. The model was used to search commercial databases of about 370,000 small molecules in total. From the approximately 1200 hits, small molecules, which were too high in molecular weight or too expensive, were excluded. From the resulting 140 hits, a group of bispyrroles proved to be interesting from a chemistry perspective and was chosen to perform a substructure search on about 600,000 small molecules. Finally a group of monopyrrole aldehydes was selected as a new class of IGPD inhibitors with activity in the low micromolar range. As this group does not fit the original pharmacophore model perfectly, it might be possible that this new group acts through a different mechanism as the original triazole inhibitors (Schweitzer et al., 2002).

A second example illustrates a screen for inhibitors of monogalactosyldiacylglycerol (MGDG) synthesis in *Arabidopsis* that used *E. coli* lipid vesicles containing recombinant MGD1 and a small molecule library with a little less than 24,000 entries. After initial screening, a new set of small molecules was put together based on chemical similarities with the hits from the first screen, which led to a selection of two small molecules: galvestine1 and galvestine2, two competitive inhibitors relative to diacylglycerol (DAG) of MGD1, MDG2, and MDG3 (Botté et al., 2011).

## Emerging target identification strategies in plant chemical biology

### Activity-based protein profiling (ABPP)

The activity-based protein profiling (ABPP) target identification strategy relies on small molecules with a so-called “warhead,” which react with residues in the active site of enzymes in an irreversible manner (van der Hoorn et al., 2011). The small molecules are attached via a linker to a functionality, such as biotin for affinity purification, or to a fluorophore for visualization. As the small molecules react with their respective target proteins to form a covalent bond, no additional cross-linking is required for further affinity purification. However, not every small molecule is capable of reacting with its target protein, therefore ABPP is only applicable for small molecules able to react with their target protein. Equally, not every protein will react with a small molecule to form a covalent bond, and thus ABPP results in a substantially less complex proteome, which facilitates a more straightforward analysis. Importantly, ABPP enables to assign activity to certain proteins, not only within the entire proteome, but also within a protein family thereby creating activity-based sub-classes. Recent examples of the use of ABPP are illustrated by studying the mode of action of the bicyclic hydantoin and several serine hydrolases (SHs) inhibitors in *Arabidopsis*.

The bicyclic hydantoin sparked the attention when it was found as a side product from synthesis efforts for syringolins (Kaschani et al., 2012a). To identify a molecular target from *Arabidopsis* cell cultures, the bicyclic hydantoin was labeled with biotin and rhodamine, and both versions were applied to either detect or pull down the protein target. An affinity purification coupled with a mass spectrometry (MS) was used to identify the glyceraldehyde 3-phosphate dehydrogenase GAPC1 and GAPC2 as targets of the bicyclic hydantoin. Both GAPC1 and GAPC2 were heterologously expressed in *E. coli*, and shown to bind the rhodamine-tagged bicyclic hydantoin in an activity-dependent manner (Kaschani et al., 2012a).

The second example employed a competitive ABPP approach to evaluate the effect of different putative SH inhibitors in *Arabidopsis* (Kaschani et al., 2012b). Competitive ABPP assesses the ability of small molecules to compete with ABPP probes. A reduced labeling by the probe in the presence of the small molecule indicates binding of the small molecule to the protein(s) under investigation. A rhodamine-tagged fluorophosphonate (FP) and a trifunctional nitrophenol phosphonate (TriNP) tagged with both rhodamine and biotin were used as ABPP probes. The main finding of the study was a differential sensitivity of different *Arabidopsis* SHs to the SH inhibitors tested (Kaschani et al., 2012b). An additional study on SHs reports the development of a paraoxon-like para-nitrophenol phosphonate activity-based probe predominantly labeling carboxylesterase12 in *Arabidopsis* (Nickel et al., 2012).

### Yeast 3-hybrid

**Figure 2:**
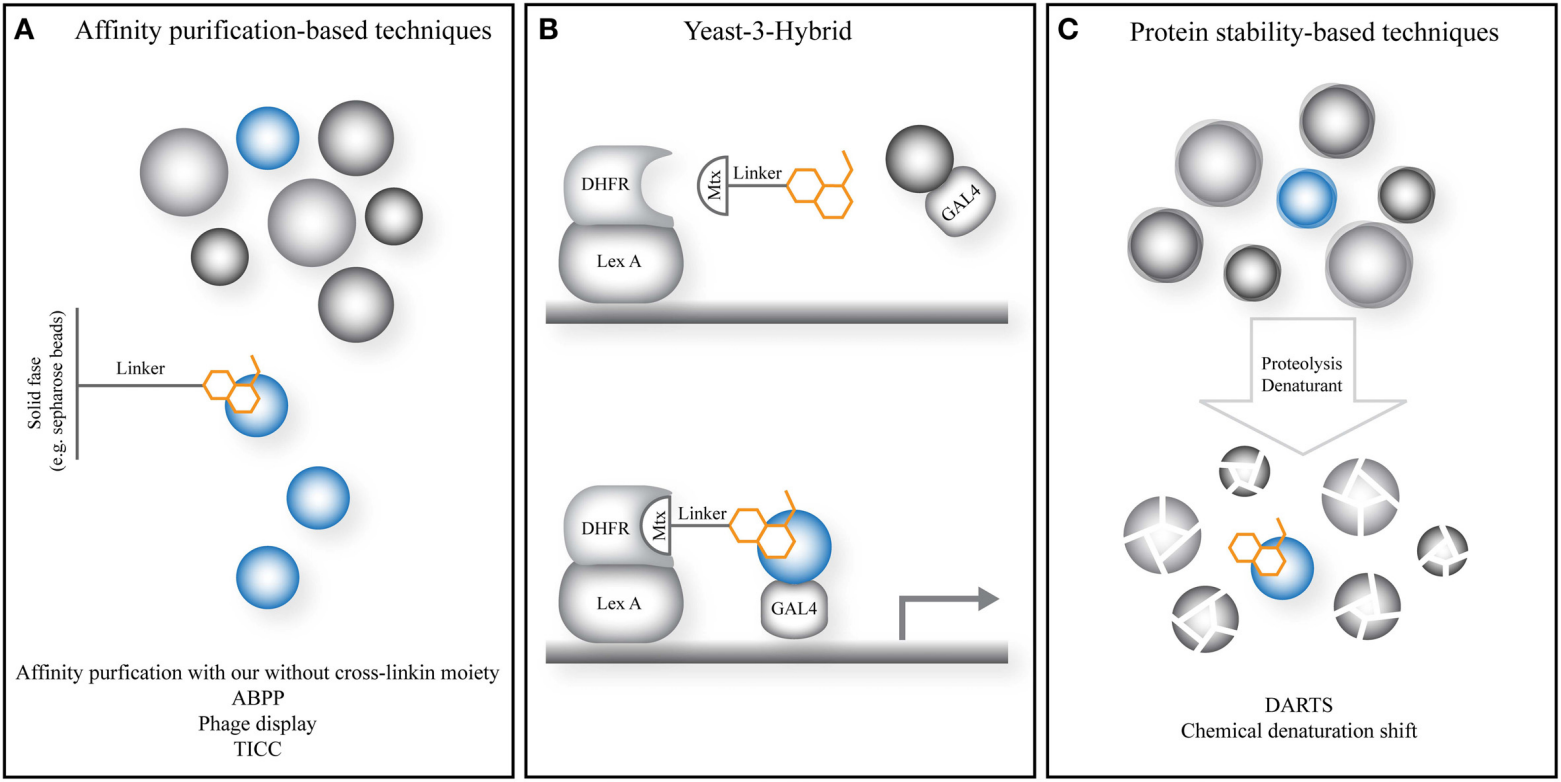
**General principle of emerging and novel target identification strategies in plant chemical biology. (A)** Strategies relying on the affinity of the small molecule to isolate the target protein from a complex mixture such as a lysate or cellular environment. **(B)** The yeast-3-hybrid approach uses the activation of a transcriptional response by bringing together a DNA-binding domain and transcriptional activator via a fusion of the small molecule of interest and a known small molecule with high affinity for a known protein target. The latter is fused to the DNA-binding domain. The small molecule probes a cDNA library fused to the transcriptional activator. **(C)** Strategies relying on increased protein stability utilize small molecules to stabilize the increased dynamics, instability and degradation upon treatments such as denaturants or proteases, preventing or slowing down target protein degradation. Gray spheres: non-target proteins; blue spheres: target protein; orange cartoon: small molecule of interest; Lex A, Lex A DNA-binding domain; Mtx, methotrexate; DHFR, dihydrofolate reductase, target protein of Mtx; GAL4, GAL4 transcriptional activator.

The yeast 3-hybrid (Y3H) approach relies on the principles of the yeast 2-hybrid (Y2H) technology, but uses a modified small molecule of interest to allow interaction between the DNA-binding domain and the transcriptional activator (Figure 2). Initially (Licitra and Liu, 1996), Y3H was based on the Y2H system using the LexA DNA-binding domain and the trans-activation domain from the bacterial protein B42 (Gyuris et al., 1993). The so-called “hook” consisted of the LexA DNA-binding domain fused to the hormone-binding domain of the rat glucocorticoid receptor. The latter binds to dexamethasone, which is part of the hybrid small molecule comprising dexamethasone and FK506, or the “bait.” Finally, the “fish” consisted of human FKBP12 fused to the transcriptional activator B42. For screening purposes, FKBP12 represents any cDNA library of choice, whereas FK506 represents the small molecule of interest.

To date, Y3H in plant chemical biology was used in an attempt to identify targets for small molecules with implications in plant defense responses (Cottier et al., 2011). The Y3H system used to this end was based on the LexA DNA-binding domain fused to dihydrofolate reductase (DHFR), which binds with high affinity to methotrexate (Mtx), and had been used previously with success (Becker et al., 2004). The hybrid ligand was composed of Mtx fused via a polyethylene glycol (PEG) linker to several small molecules, namely jasmonic acid (JA), ABA, compound 8 (cpd 8), cucurbic acid (CA), cucurbic acid methylester (CAMe) and 2,6 dihydroxybenzoic acid (6OH-SA). The “fish” was a collection of cDNA libraries from wounded or pathogen infected leaves and inflorescence from *Arabidopsis*, fused to the GAL4 transcriptional activator (Cottier et al., 2011). Although no targets were identified for Mtx-ABA and Mtx-JA, and no interaction could be shown when the known ABA and JA receptors were expressed as “fish,” potential target proteins were identified for the other small molecules. This study validates Y3H as a target identification strategy for plant chemical biology.

The Y3H technique has a few notable advantages because it allows screening for small molecule-protein interactions *in vivo*, correction for low abundant proteins, easy identification of the target protein(s) or even interacting protein domains and, additionally, detection of essential gene products, and even straightforward characterization of an entire protein family as the target of a small molecule. However, although Y3H is an *in vivo* method, the ability of the protein to bind the small molecule of interest is assessed out of its biological context and one protein at a time, and therefore is less suited when small molecules require more than one protein to bind or the proper biological context. Moreover, proteins not able to translocate to the yeast nucleus, such as trans-membrane or membrane-associated proteins cannot be screened; also the fact that the small molecules need to be modified, and the occurrence of multi-drug resistance in yeast can pose a problem. Variants of the Y3H system have been described (Ziegler et al., 2013), but have not found an implementation in plant chemical biology yet.

An improved version of Y3H screening is based on covalent labeling of SNAP-tag fusion proteins (Chidley et al., 2011). The SNAP-tag is based on the human O^6^-alkylguanine-DNA alkyltransferase that will covalently attach the alkyl group of its substrate to one of its cysteine residues. As its substrate specificity is not so high, it can also accept O^6^-benzylguanine (BG) as a substrate (Keppler et al., 2003). The Y3H is modified in such a way that the DNA-binding domain LexA is fused to a SNAP-tag, and the small molecule of interest is derivatized with BG (Chidley et al., 2011). The improved Y3H approach includes first, the use of a triple mutant for broad-spectrum drug transporters. Second, false-positives are eliminated by a negative selection using 5-fluoroorotic acid in the absence of the modified small molecule, and later colonies are grown both in the presence and absence of the modified compound to score for specific interactions. Additionally, growth of colonies in the presence of the “bait” and an excess of the free, unmodified small molecule could potentially pinpoint colonies expressing a specific target, because the free small molecule will out-compete the “bait,” thereby preventing further growth or reporter expression (Licitra and Liu, 1996). The SNAP-tag can also be combined with a GST-tag and thus, the GST-SNAP-tagged fusion protein can readily be used in affinity purification approaches with the same modified small molecules (Chidley et al., 2011).

Concerning synthesis of the modified small molecule for Y3H, a recent study evaluated the length and nature of the linker (Tran et al., 2013). An important conclusion of the use of a triazole-containing linker opposed to a PEG linker was the lower background growth of yeast in the presence of a negative control, and thus a decreased amount of possible false positives.

### Affinity-based technologies

A much less explored target identification strategy in plant chemical biology is affinity purification. Typically, a derivatized small molecule is generated consisting of a selectivity function, which is the small molecule of interest, bound via a linker moiety to a tag such as biotin, which allows target isolation from a complex mixture. Incubation of an appropriate lysate with the modified compound, immobilization on a solid support, and subsequent washing of unbound proteins enable isolation of target proteins for liquid chromatography and MS analysis (Figure 2). As such, the general principle of this approach is shared with ABPP.

Crucial for derivatization is structure activity relation (SAR) analysis. SAR analysis involves testing of a collection of analogs of the original small molecule to assess which functional groups and moieties are essential for its activity. SAR analysis is not only important for derivatization, but can also provide crucial information about the mode of action of small molecules. A good illustration of the latter is sirtinol, identified as an inhibitor of sirtuin deacetylases in yeast and human cells (Grozinger et al., 2001). Sirtinol in *Arabidopsis* was characterized as an enhancer of auxin signaling, and was found to bind and inhibit SIRTINOL RESISTANT1 (SIR1), a negative regulator of auxin signaling upstream of the *Aux/IAA* genes. *SIR1* and other *SIR* genes encode proteins involved in molybdopterin biosynthesis, and the incorporation of molybdenum to form molybdenum cofactor (moco), an essential cofactor in for example aldehyde oxidases (Zhao et al., 2003). SAR analysis predicted that sirtinol can be hydrolyzed into 2-hydroxy-1-naphthaldehyde (HNA). HNA is subsequently converted by a moco-containing aldehyde oxidase into 2-hydroxy-1-naphtoic acid (HNC). The latter is an active auxin analog, hence explaining the sirtinol-induced phenotypes (Dai et al., 2005).

An important advantage of affinity purification is the ability to probe for target proteins of any molecular process of interest, in other words, any small molecule can be potentially used, and does not require activity toward the target in contrast to ABPP. It provides the possibility to uncover the small molecule interactome, thus not only the main target, but also “off-targets,” might contribute to the observed phenotype. The latter implies that specific readouts should be available to distinguish the target of interest from off-targets in the validation procedure. Additionally, affinity purification yields data on potential targets as well as biochemical proof of binding, provided proper controls are included.

A variant of affinity purification consists of the incorporation of a cross-linking moiety, thus giving rise to a tri-functional probe. These so-called “capture compounds” consist of a selectivity function, which is the small molecule of interest, a reactivity function, which is the cross-linking moiety, and a sorting function (such as biotin) (Köster et al., 2007; Fischer et al., 2010, 2011). The cross-linking moiety usually is activated by UV-light, thereby forming covalent bonds with proteins in close proximity. As the small molecule-target interaction is secured by a covalent bond, washing can be stringent, removing unspecific binders; hence, incorporating a cross-linking moiety might solve problems with weak interactions and low abundant or less accessible protein targets. Crucial for both affinity purifications and procedures involving cross-linking moieties are proper controls to distinguish “true” targets from non-specific interactors. Preferably an inactive analog of the small molecule modified in the same way is used, but it should be noted that inactivity *in vivo* does not necessarily mean it will not bind the target protein in a lysate, because inactivity might be due to altered uptake. In addition, the “interactome” for the solid phase used (such as streptavidin-coated beads), or the linker with biotin alone might serve as an essential background list. Equally, multiple repeats with different probes can distinguish targets from background signals in a statistical manner. Competition experiments with unmodified compounds may reveal specific binders from non-specific binders, and serve as an essential control. However, in order to detect a competition with the unmodified small molecule, quantification of eluted proteins is required. To this end, stable isotope labeling with amino acids in cell culture (SILAC) (Ong et al., 2002, 2009) can be used or other forms of differential labeling of peptides or proteins (Gant-Branum et al., 2009; Collier and Muddiman, 2012). In short, two samples are prepared and differentially labeled (e.g., heavy and light) according to the SILAC protocol. Both samples are differentially treated: one with the probe only, the other with the probe and free small molecule in competition. When the concentration of the free small molecule is high enough, and when the affinity toward the target is higher than that of the modified small molecule, specific targets should not be retained after pull-down. Subsequent combination of both samples in a 1:1 ratio and MS analysis results in a distinguishable peptide pair originating from the different samples because they only differ by their isotopic mass difference. Non-specific binders should be represented by peptides of equal intensity for both samples, opposed to peptides representing specific binders, because their intensity should be much lower due to the imposed competition with the free small molecule (Ong et al., 2009). Additional issues might arise when the target protein is low abundant or of hydrophobic nature.

One of the first examples of compound photo-affinity labeling in plant research concerns the modification of atrazine with a photo-reactive azido group. The subsequent azido-atrazine was radioactively labeled to allow detection of covalently bound polypeptides on a polyacrylamide gel (Pfister et al., 1981). Although atrazine is a well-characterized inhibitor of photosystem II reactions, and azido-atrazine was shown to act in a very similar way, target identification stops at the level of radioactively labeled polypeptides of 32–34 kDa on a polyacrylamide gel, be it from purified chloroplast thylakoids.

A second example is given by efforts to identify the molecular target of the small molecule responsible for nyctinastic leaf movement of *Albizzia saman* (Nakamura et al., 2008). The tri-functional probe consisted of the small molecule of interest, a jasmonate glucoside, benzophenone as the reactivity function and biotin as the selectivity function. Additionally, an inactive enantiomeric analog was modified in the same way, serving as a control (Nakamura et al., 2008). Both probes were activated at 365 nm, and SDS-PAGE analysis revealed a differential band, which disappeared using the unmodified small molecule as competitor.

A third example concerns the biotin-labeled photoaffinity castasterone (BPCS) (Kinoshita et al., 2005). This report shows the ability of castasterone, an active BR (Vriet et al., 2013), to bind the BR receptor BR-INSENSITIVE1 (BRI1). The binding site is mapped to an island domain in between leucine-rich repeat 21 (LRR21) and LRR22 of the extracellular domain of BRI1. This observation was later confirmed by structural data of BRI1 in complex with brassinolide (Hothorn et al., 2011; She et al., 2011). Until now to our knowledge no examples exist in plant research of an affinity purification approach without covalent binding to the target protein.

In order to circumvent the bio-availability problems that are likely to arise with biotin tags or fluorophores attached to the small molecule, a two-step labeling of small molecules was optimized in *Arabidopsis* using so-called mini-tags, based on azide and alkyn functional groups (Kaschani et al., 2009). The well-known cysteine-protease inhibitor E64 was used to study and establish the two-step labeling technique. Because E64 is a covalent inhibitor of its targets, the two-step labeling consists of a first modification of E64 with a mini-tag, minimizing interference of the tag on E64 activity and bioavailability. After incubation of the sample with modified E64, a second step attaches biotin modified with the appropriate mini-tag in a click-chemistry reaction, allowing subsequent purification and detection of the target protein (Kaschani et al., 2009). One of the main advantages is the ability to label *in vivo*, which, in the explained setup is not possible for small molecules that do not bind covalently to their target protein. This problem can be solved by introducing a photo-activatable group together with a mini-tag, which allows cross-linking *in vivo*, with subsequent preparation of the lysate and attaching biotin for affinity purification.

Although *in situ* proteome profiling with a small molecule modified for photo-cross-linking has the advantage of identifying target proteins in the proper biological context, care should be taken with possible effects of UV irradiation on the proteome. Certainly when exposure lasts for several minutes, damage might be induced, which eventually might compromise the final protein target list.

In order to perform *in situ* proteome profiling, the small molecule of choice should be modified with a photo-cross-linking group, together with a group that allows additional bioorthogonal modification, usually done with a clickable group. In this way, after cross-linking in live cells, a group for affinity purification or visualization can be added afterward. The latter option allows the usage of a technique called fluorescence difference in two-dimensional gel electrophoresis (FITGE). This method uses two samples, one labeled with the active small molecule, the other with an inactive form or other control, and labels both samples with a different fluorophore. This differential labeling allows detection of both samples together on a 2D SDS-PAGE gel. Spots that are labeled by both fluorophores are probable because of unspecific binding events, whereas spots only labeled with the fluorophore attached to the active small molecule are potential hits that can be identified by subsequent MS approaches (Park et al., 2012). Differences between lysates and live cells as starting material were reported, in which live cells might likely provide more reliable target identification and can be even a requirement to detect the main target.

### Phage display

The phage display strategy relies on whole, fragmented cDNA or random peptide sequences translationally fused to the phage coat protein, so that the peptides are displayed on the outside. An immobilized small molecule can retain the peptides that bind to the small molecule. A subsequent bacterial infection allows the identification of selected peptides. A recent example in plants used a quartz-crystal microbalance (QCM) biosensor in combination with T7 phage display and the receptor-ligand contacts (RELIC) bioinformatics server to identify binding sites for the BR biosynthesis inhibitor brassinazole (Brz2001) in the cytochrome P450 enzyme DWARF4 (DWF4) that catalyzes the rate-limiting hydroxylation of the C22 position in the BR biosynthesis (Asami et al., 2000; Sekimata et al., 2001; Takakusagi et al., 2013; Vriet et al., 2013). The QCM measures voltage-induced crystal vibrations on a gold electrode, which will decrease as the overall mass on the gold electrode increases. Takakusagi et al. (2013) used a modified version of Brz2001, which forms a monolayer on the gold electrode. The QCM-measured vibrations will decrease as peptides bind the immobilized small molecule. A random 15-mer peptide library was incubated with the Brz2001-covered gold electrode, which resulted in the identification of 34 peptides. Subsequent use of the RELIC bioinformatics platform (Mandava et al., 2004) detected within the 34 selected peptides a subset of amino acids potentially involved in small molecule binding that map to a potential disordered loop of DWF4 (Takakusagi et al., 2013).

Given the possibility to modify the small molecule of interest, and the ability to coat the gold electrode, this technique allows a quick assessment of possible target proteins. It allows a coverage of the proteome without the possibility of missing out on low abundant proteins due to the easy amplification of the signal by bacterial infection. It is less suited for interactions requiring post-translational modifications, very hydrophobic peptides, and protein-small molecule interactions, for which several amino acid residues involved in binding are scattered across the protein primary sequence. When the small molecule only binds the appropriate amino acid residues form a binding pocket after protein folding, phage display with a random small peptide library will likely not work. Phage display with entire proteins might solve the problem, but will select any hydrophobic protein, besides the possibility that the protein might not fold properly.

## Label-free compound-based technologies

An obvious drawback of affinity purification is the requirement for “taggable” positions on the small molecule of interest. In addition, the small molecule should still be active with at least part of the intended modification (such as the linker), because the modification might hinder proper binding of the small molecule to its targets. Efficient isolation of the target is also dependent on the affinity of the small molecule, because low-affinity interactions might be lost during washing. Therefore, rather gentle washing conditions should be used, which have the disadvantage of generating extensive lists of possible target proteins. In an effort to solve these problems, approaches that do not require labeled small molecules are being developed.

### Chemical denaturation shift

One way of testing ligand interactions is by measuring protein stability, which depends on a number of factors, including temperature, denaturants and ligand binding. An increase in protein stability, and thus denaturing conditions, to higher temperatures is indicative of ligand binding. Classically, this is measured by fluorescence or differential scanning calorimetry (Straume and Freire, 1992; Lo et al., 2004). However, because estimations on binding affinities require prior knowledge on enthalpy and heat capacity of protein denaturation and ligand binding, such an approach is not suited for high throughput screening, because binding thermodynamics are yet unknown for small molecules in a library. Additionally, the rank order given to small molecule interactors based on the induced shift in protein denaturation temperature (T_m_) is not necessarily the same at the physiological temperature and the measured T_m_. An alternative approach was proposed based on a chemical denaturation shift (Schön et al., 2013), in which instead of measuring T_m_, the increase in concentration of a denaturant is measured required to denature the protein in the presence of its ligand. Although the study is an optimization and proof of concept of the chemical denaturation shift approach, it illustrates the usability to provide proof of ligand binding, and is additionally suited for high-throughput screening setups in a reverse chemical genetics strategy.

### Target identification by chromatographic co-elution (TICC)

The main disadvantage of labeling small molecules with any tag, is the possibility of selecting against small molecules or natural products that do not allow any modification (i.e., they lose biological activity altogether). To this end, target identification strategies using unmodified small molecules are being developed. One example of such a strategy is the TICC technology (Chan et al., 2012). The idea is to look for a shift in the retention time of the small molecule of interest in a complex protein mixture compared to the small molecule alone during non-denaturing high-performance liquid chromatography. The shift in retention time would be indicative of binding to a particular protein target, which can be identified by further deconvolving the fraction in which the small molecule elutes by additional complementing and orthogonal fractionations. Key to the success of this approach is the ability to separate free from protein-bound ligands (Chan et al., 2012).

### Drug affinity-responsive target stability (DARTS)

The DARTS approach takes advantage of the stabilization of a protein target upon small molecule binding, thereby rendering the protein less susceptible to proteolytic digestion (Lomenick et al., 2009, 2011). This idea also formed the basis for the chemical denaturation shift approach (Pace and McGrath, 1980). DARTS can be used to confirm a certain small molecule-protein interaction by specifically evaluating proteolytic digestion via western blotting, but equally can be used to evaluate possible new small molecule-protein interactions by looking at entire lysates. Although the latter situation might result in visibly stabilized targets when high abundant, low abundant target proteins might not be readily visible on gel (Lomenick et al., 2009).

Both DARTS and TICC share some important advantages. First they are label free, require no derivatization and use the original small molecule. This is not only important in terms of small molecule tolerance toward modification, but also saves time, as SAR analysis can be limited. A second important advantage is their independency of any protein nature, mode of action or model system. Both techniques solely rely on affinity of the small molecule for its target protein. The latter also dictates an inherent weakness: interactions of lower affinity might be missed. Both techniques have their specific weaknesses too. Whereas membrane proteins remain challenging for TICC, DARTS is not applicable to any protein, because some proteins are more resistant to digestion and might be missed. In addition, the small molecule might interact in such a way that digestion of the protein is not, or hardly affected.

## Future perspectives

Traditionally, chemical biology approaches have a strong background in pharmaceutical and agricultural fields, whereas basic research lags behind, certainly in plant biology. Over the recent years though, plant biology has witnessed an increasing interest in chemical biology approaches for processes such as endomembrane trafficking, hormonal signaling and primary and secondary metabolism (Hicks and Raikhel, 2012; Mishev et al., 2013; Tresch, 2013; Ma and Robert, 2014). Depending on which aspect of plant biology the small molecule of choice is affecting, and what the intended use will be, knowledge on the interactome of the small molecule might be essential. As the induced phenotype is only the sum of the individual targets affected, a deconvolution of the phenotype toward the individual contributions of the affected targets is of paramount importance. To this end, current commonly used target identification strategies in plant biology fall short. Therefore, an evolution toward more biochemical and alternative strategies is required. Surprisingly, one of the most successful target identification strategies in animal research is much less used in plant research: affinity purification. Although the technique has important shortcomings such as the need for small molecule modification and the preference for abundant soluble protein targets, it is one of the few strategies capable of revealing the small molecule interactome. Several variants exist of the basic affinity-based pull-down principle to accommodate for shortcomings of the technique, and their implementation in fundamental plant biology research as well as in a more commercial research environment should spur our understanding of dynamic cellular mechanisms. In addition, development of affinity purification-based approaches in combination with other well-established techniques might provide additional dimensions to the interactome resulting from classic affinity-based setups. One such example is Chem-seq, in which a combination of affinity purification and chromatin immunoprecipitation (ChIP) technology can provide new insights into the role of small molecules at a genome-wide level (Anders et al., 2014).

Other approaches that are more established in other systems than in plants, besides the ones mentioned in this review, might find their way into plant biology as well. A possible example could be multi-copy suppression profiling, in which the central idea relies on increased tolerance toward the small molecule when the target protein is present in higher copy numbers (Ziegler et al., 2013). Similarly to EMS screens, overexpression line collections such as activation-tag collections could be screened for more tolerance toward small molecules. Problems with gene redundancy and lethality would be overcome, and identification of the potential target should be simple. Equally, one overexpression line could be used to screen an entire collection of small molecules for more tolerance. In addition, adaptation of the Cellular Thermal Shift Assay (CETSA) (Martinez Molina et al., 2013) for target identification purposes might be the onset toward a relatively easy strategy to identify possible target proteins without the need of small molecule modification. The technique relies on increased stability of the target protein in the presence of the small molecule at higher temperatures, according to a similar principle as DARTS and the chemical denaturation shift. Moreover, this approach has proved to be successful at the cellular and even tissue level (Martinez Molina et al., 2013).

Finally, the choice for a particular target identification strategy greatly depends on the aim of the study and available resources, still considering that several complementary approaches to prove protein target binding will only make the study stronger. Although initial efforts to setup an affinity purification target identification approach are greater compared to for example resistance screens, affinity purification has important advances over the well-established target identification strategies in plant biology. Therefore, plant biology can only benefit from adapting affinity-based target identification approaches in future chemical biology projects.

### Conflict of interest statement

The authors declare that the research was conducted in the absence of any commercial or financial relationships that could be construed as a potential conflict of interest.

## References

[B1] Anders L. Guenther M. G. Qi J. Fan Z. P. Marineau J. J. Rahl P. B. (2014). Genome-wide localization of small molecules. Nat. Biotechnol. 32, 92–96 10.1038/nbt.2776 24336317 PMC4189815

[B2] Asami T. Min Y. K. Nagata N. Yamagishi K. Takatsuto S. Fujioka S. (2000). Characterization of brassinazole, a triazole-type brassinosteroid biosynthesis inhibitor. Plant Physiol. 123, 93–99 10.1104/pp.123.1.93 10806228 PMC58985

[B3] Becker F. Murthi K. Smith C. Come J. Costa-Roldán N. Kaufmann C. (2004). A three-hybrid approach to scanning the proteome for targets of small molecule kinase inhibitors. Chem. Biol. 11, 211–223 10.1016/j.chembiol.2004.02.001 15123283

[B4] Botté C. Y. Deligny M. Roccia A. Bonneau A.-L. Saïdani N. Hardré H. (2011). Chemical inhibitors of monogalactosyldiacylglycerol synthases in *Arabidopsis thaliana*. Nat. Chem. Biol. 7, 834–842 10.1038/nchembio.658 21946275

[B5] Chan J. N. Y. Vuckovic D. Sleno L. Olsen J. B. Pogoutse O. Havugimana P. (2012). Target identification by chromatographic co-elution: monitoring of drug-protein interactions without immobilization or chemical derivatization. Mol. Cell. Proteomics 11:M111.016642 10.1074/mcp.M111.016642 22357554 PMC3394955

[B6] Chidley C. Haruki H. Pedersen M. G. Muller E. Johnsson K. (2011). A yeast-based screen reveals that sulfasalazine inhibits tetrahydrobiopterin biosynthesis. Nat. Chem. Biol. 7, 375–383 10.1038/nchembio.557 21499265

[B7] Collier T. S. Muddiman D. C. (2012). Analytical strategies for the global quantification of intact proteins. Amino Acids 43, 1109–1117 10.1007/s00726-012-1285-z 22821264

[B8] Cottier S. Mönig T. Wang Z. Svoboda J. Boland W. Kaiser M. (2011). The yeast three-hybrid system as an experimental platform to identify proteins interacting with small signaling molecules in plant cells: potential and limitations. Front. Plant Sci. 2:101 10.3389/fpls.2011.00101 22639623 PMC3355722

[B9] Dai X. Hayashi K.-i., Nozaki H. Cheng Y. Zhao Y. (2005). Genetic and chemical analyses of the action mechanisms of sirtinol in *Arabidopsis*. Proc. Natl. Acad. Sci. U.S.A. 102, 3129–3134 10.1073/pnas.0500185102 15710899 PMC549487

[B10] De Rybel B. Audenaert D. Vert G. Rozhon W. Mayerhofer J. Peelman F. (2009). Chemical inhibition of a subset of *Arabidopsis thaliana* GSK3-like kinases activates brassinosteroid signaling. Chem. Biol. 16, 594–604 10.1016/j.chembiol.2009.04.008 19549598 PMC4854203

[B11] De Rybel B. Audenaert D. Xuan W. Overvoorde P. Strader L. C. Kepinski S. (2012). A role for the root cap in root branching revealed by the non-auxin probe naxillin. Nat. Chem. Biol. 8, 798–805 10.1038/nchembio.1044 22885787 PMC3735367

[B12] Fischer J. J. Graebner O. Dreger M. Glinski M. Baumgart S. Koester H. (2011). Improvement of capture compound mass spectrometry technology (CCMS) for the profiling of human kinases by combination with 2D LC-MS/MS. J. Biomed. Biotechnol. 2011:850589 10.1155/2011/850589 21941435 PMC3176445

[B13] Fischer J. J. Michaelis S. Schrey A. K. Graebner nee Baessler O. Glinski M. Dreger M. (2010). Capture compound mass spectrometry sheds light on the molecular mechanisms of liver toxicity of two Parkinson drugs. Toxicol. Sci. 113, 243–253 10.1093/toxsci/kfp236 19783845

[B14] Gant-Branum R. L. Kerr T. J. McLean J. A. (2009). Labeling strategies in mass spectrometry-based protein quantitation. Analyst 134, 1525–1530 10.1039/b904643g 20448914

[B15] Grozinger C. M. Chao E. D. Blackwell H. E. Moazed D. Schreiber S. L. (2001). Identification of a class of small molecule inhibitors of the sirtuin family of NAD-dependent deacetylases by phenotypic screening. J. Biol. Chem. 276, 38837–38843 10.1074/jbc.M106779200 11483616

[B16] Gyuris J. Golemis E. Chertkov H. Brent R. (1993). Cdi1, a human G1 and S phase protein phosphatase that associates with Cdk2. Cell 75, 791–803 10.1016/0092-8674(93)90498-F 8242750

[B17] He W. Brumos J. Li H. Ji Y. Ke M. Gong X. (2011). A small-molecule screen identifies l-kynurenine as a competitive inhibitor of TAA1/TAR activity in ethylene-directed auxin biosynthesis and root growth in *Arabidopsis*. Plant Cell 23, 3944–3960 10.1105/tpc.111.089029 22108404 PMC3246337

[B18] Hicks G. R. Raikhel N. V. (2012). Small molecules present large opportunities in plant biology. Annu. Rev. Plant Biol. 63, 261–282 10.1146/annurev-arplant-042811-105456 22404475

[B19] Hothorn M. Belkhadir Y. Dreux M. Dabi T. Noel J. P. Wilson I. A. (2011). Structural basis of steroid hormone perception by the receptor kinase BRI1. Nature 474, 467–471 10.1038/nature10153 21666665 PMC3280218

[B20] Kaschani F. Clerc J. Krahn D. Bier D. Hong T. N. Ottmann C. (2012a). Identification of a selective, activity-based probe for glyceraldehyde 3-phosphate dehydrogenases. Angew. Chem. Int. Edit. 51, 5230–5233 10.1002/anie.201107276 22489074

[B21] Kaschani F. Nickel S. Pandey B. Cravatt B. F. Kaiser M. van der Hoorn R. A. L. (2012b). Selective inhibition of plant serine hydrolases by agrochemicals revealed by competitive ABPP. Bioorg. Med. Chem. 20, 597–600 10.1016/j.bmc.2011.06.040 21764588 PMC3634566

[B22] Kaschani F. Verhelst S. H. L. van Swieten P. F. Verdoes M. Wong C.-S. Wang Z. (2009). Minitags for small molecules: detecting targets of reactive small molecules in living plant tissues using ‘click chemistry.’ Plant J. 57, 373–385 10.1111/j.1365-313X.2008.03683.x 18786180

[B23] Keppler A. Gendreizig S. Gronemeyer T. Pick H. Vogel H. Johnsson K. (2003). A general method for the covalent labeling of fusion proteins with small molecules *in vivo*. Nat. Biotechnol. 21, 86–89 10.1038/nbt765 12469133

[B24] Kinoshita T. Caño-Delgado A. Seto H. Hiranuma S. Fujioka S. Yoshida S. (2005). Binding of brassinosteroids to the extracellular domain of plant receptor kinase BRI1. Nature 433, 167–171 10.1038/nature03227 15650741

[B25] Köster H. Little D. P. Luan P. Muller R. Siddiqi S. M. Marappan S. (2007). Capture compound mass spectrometry: a technology for the investigation of small molecule protein interactions. Assay Drug Dev. Technol. 5, 381–390 10.1089/adt.2006.039 17638538

[B26] Kumari S. van der Hoorn R. A. L. (2011). A structural biology perspective on bioactive small molecules and their plant targets. Curr. Opin. Plant Biol. 14, 480–488 10.1016/j.pbi.2011.06.003 21803639

[B27] Licitra E. J. Liu J. O. (1996). A three-hybrid system for detecting small ligand–protein receptor interactions. Proc. Natl. Acad. Sci. U.S.A. 93, 12817–12821 10.1073/pnas.93.23.12817 8917502 PMC24003

[B28] Lo M.-C. Aulabaugh A. Jin G. Cowling R. Bard J. Malamas M. (2004). Evaluation of fluorescence-based thermal shift assays for hit identification in drug discovery. Anal. Biochem. 332, 153–159 10.1016/j.ab.2004.04.031 15301960

[B29] Lomenick B. Hao R. Jonai N. Chin R. M. Aghajan M. Warburton S. (2009). Target identification using drug affinity responsive target stability (DARTS). Proc. Natl. Acad. Sci. U.S.A. 106, 21984–21989 10.1073/pnas.0910040106 19995983 PMC2789755

[B30] Lomenick B. Olsen R. W. Huang J. (2011). Identification of direct protein targets of small molecules. ACS Chem. Biol. 6, 34–46 10.1021/cb100294v 21077692 PMC3031183

[B31] Lounkine E. Keiser M. J. Whitebread S. Mikhailov D. Hamon J. Jenkins J. L. (2012). Large-scale prediction and testing of drug activity on side-effect targets. Nature 486, 361–367 10.1038/nature11159 22722194 PMC3383642

[B32] Ma Q. Robert S. (2014). Auxin biology revealed by small molecules. Physiol. Plant 151, 25–42 10.1111/ppl.12128 24252105

[B33] Ma Y. Szostkiewicz I. Korte A. Moes D. Yang Y. Christmann A. (2009). Regulators of PP2C phosphatase activity function as abscisic acid sensors. Science 324, 1064–1068 10.1126/science.1172408 19407143

[B34] Mandava S. Makowski L. Devarapalli S. Uzubell J. Rodi D. J. (2004). RELIC–a bioinformatics server for combinatorial peptide analysis and identification of protein-ligand interaction sites. Proteomics 4, 1439–1460 10.1002/pmic.200300680 15188413

[B35] Martinez Molina D. Jafari R. Ignatushchenko M. Seki T. Larsson E. A. Dan C. (2013). Monitoring drug target engagement in cells and tissues using the cellular thermal shift assay. Science 341, 84–87 10.1126/science.1233606 23828940

[B36] Mishev K. Dejonghe W. Russinova E. (2013). Small molecules for dissecting endomembrane trafficking: a cross-systems view. Chem. Biol. 20, 475–486 10.1016/j.chembiol.2013.03.009 23601636

[B37] Mori I. Fonné-Pfister R. Matsunaga S.-i., Tada S. Kimura Y. Iwasaki G. (1995). A novel class of herbicides. Specific inhibitors of imidazoleglycerol phosphate dehydratase. Plant Physiol. 107, 719–723 10.1104/pp.107.3.719 12228396 PMC157187

[B38] Nakamura Y. Miyatake R. Ueda M. (2008). Enantiodifferential approach for the detection of the target membrane protein of the jasmonate glycoside that controls the leaf movement of *Albizzia saman*. Angew. Chem. Int. Edit. 47, 7289–7292 10.1002/anie.200801820 18683266

[B39] Nickel S. Kaschani F. Colby T. van der Hoorn R. A. L. Kaiser M. (2012). A *para*-nitrophenol phosphonate probe labels distinct serine hydrolases of Arabidopsis. Bioorg. Med. Chem. 20, 601–606 10.1016/j.bmc.2011.06.041 21763150

[B40] Noutoshi Y. Okazaki M. Kida T. Nishina Y. Morishita Y. Ogawa T. (2012). Novel plant immune-priming compounds identified via high-throughput chemical screening target salicylic acid glucosyltransferases in *Arabidopsis*. Plant Cell 24, 3795–3804 10.1105/tpc.112.098343 22960909 PMC3480303

[B41] Ong S.-E. Blagoev B. Kratchmarova I. Kristensen D. B. Steen H. Pandey A. (2002). Stable isotope labeling by amino acids in cell culture, SILAC, as a simple and accurate approach to expression proteomics. Mol. Cell. Proteomics 1, 376–386 10.1074/mcp.M200025-MCP200 12118079

[B42] Ong S.-E. Schenone M. Margolin A. A. Li X. Do K. Doud M. K. (2009). Identifying the proteins to which small-molecule probes and drugs bind in cells. Proc. Natl. Acad. Sci. U.S.A. 106, 4617–4622 10.1073/pnas.0900191106 19255428 PMC2649954

[B43] Pace C. N. McGrath T. (1980). Substrate stabilization of lysozyme to thermal and guanidine hydrochloride denaturation. J. Biol. Chem. 255, 3862–3865 7372654

[B44] Park J. Oh S. Park S. B. (2012). Discovery and target identification of an antiproliferative agent in live cells using fluorescence difference in two-dimensional gel electrophoresis. Angew. Chem. Int. Edit. 51, 5447–5451 10.1002/anie.201200609 22489056

[B45] Park S.-Y. Fung P. Nishimura N. Jensen D. R. Fujii H. Zhao Y. (2009). Abscisic acid inhibits type 2C protein phosphatases via the PYR/PYL family of START proteins. Science 324, 1068–1071 10.1126/science.1173041 19407142 PMC2827199

[B46] Pfister K. Steinback K. E. Gardner G. Arntzen C. J. (1981). Photoaffinity labeling of an herbicide receptor protein in chloroplast membranes. Proc. Natl. Acad. Sci. U.S.A. 78, 981–985 10.1073/pnas.78.2.981 16592984 PMC319929

[B47] Rojas-Pierce M. Titapiwatanakun B. Sohn E. J. Fang F. Larive C. K. Blakeslee J. (2007). *Arabidopsis* P-glycoprotein19 participates in the inhibition of gravitropism by gravacin. Chem. Biol. 14, 1366–1376 10.1016/j.chembiol.2007.10.014 18096505

[B48] Santiago J. Dupeux F. Round A. Antoni R. Park S.-Y. Jamin M. (2009). The abscisic acid receptor PYR1 in complex with abscisic acid. Nature 462, 665–668 10.1038/nature08591 19898494

[B49] Schön A. Brown R. K. Hutchins B. M. Freire E. (2013). Ligand binding analysis and screening by chemical denaturation shift. Anal. Biochem. 443, 52–57 10.1016/j.ab.2013.08.015 23994566 PMC3809086

[B50] Schweitzer B. A. Loida P. J. CaJacob C. A. Chott R. C. Collantes E. M. Hegde S. G. (2002). Discovery of imidazole glycerol phosphate dehydratase inhibitors through 3-D database searching. Bioorg. Med. Chem. Lett. 12, 1743–1746 10.1016/S0960-894X(02)00283-4 12067551

[B51] Sekimata K. Kimura T. Kaneko I. Nakano T. Yoneyama K. Takeuchi Y. (2001). A specific brassinosteroid biosynthesis inhibitor, Brz2001: evaluation of its effects on *Arabidopsis*, cress, tobacco, and rice. Planta 213, 716–721 10.1007/s004250100546 11678275

[B52] She J. Han Z. Kim T.-W. Wang J. Cheng W. Chang J. (2011). Structural insight into brassinosteroid perception by BRI1. Nature 474, 472–476 10.1038/nature10178 21666666 PMC4019668

[B53] Straume M. Freire E. (1992). Two-dimensional differential scanning calorimetry: simultaneous resolution of intrinsic protein structural energetics and ligand binding interactions by global linkage analysis. Anal. Biochem. 203, 259–268 10.1016/0003-2697(92)90311-T 1416022

[B54] Surpin M. Rojas-Pierce M. Carter C. Hicks G. R. Vasquez J. Raikhel N. V. (2005). The power of chemical genomics to study the link between endomembrane system components and the gravitropic response. Proc. Natl. Acad. Sci. U.S.A. 102, 4902–4907 (Erratum *Proc. Natl. Acad. Sci. U.S.A* 102, 10752). 10.1073/pnas.0500222102 15772170 PMC555711

[B55] Takakusagi Y. Manita D. Kusayanagi T. Izaguirre-Carbonell J. Takakusagi K. Kuramochi K. (2013). Mapping a disordered portion of the Brz2001-binding site on a plant monooxygenase, DWARF4, using a quartz-crystal microbalance biosensor-based T7 phage display. Assay Drug Dev. Technol. 11, 206–215 10.1089/adt.2012.478 23514038

[B56] Tóth R. van der Hoorn R. A. L. (2010). Emerging principles in plant chemical genetics. Trends Plant Sci. 15, 81–88 10.1016/j.tplants.2009.11.005 20036182

[B57] Tran F. Odell A. V. Ward G. E. Westwood N. J. (2013). A modular approach to triazole-containing chemical inducers of dimerisation for yeast three-hybrid screening. Molecules 18, 11639–11657 10.3390/molecules180911639 24064457 PMC4031444

[B58] Tresch S. (2013). Strategies and future trends to identify the mode of action of phytotoxic compounds. Plant Sci. 212, 60–71 10.1016/j.plantsci.2013.08.005 24094055

[B59] van der Hoorn R. A. L. Colby T. Nickel S. Richau K. H. Schmidt J. Kaiser M. (2011). Mining the active proteome of *Arabidopsis thaliana*. Front. Plant Sci. 2:89 10.3389/fpls.2011.00089 22639616 PMC3355598

[B60] Vriet C. Russinova E. Reuzeau C. (2013). From squalene to brassinolide: the steroid metabolic and signaling pathways across the plant kingdom. Mol. Plant 6, 1738–1757 10.1093/mp/sst096 23761349

[B61] Walsh T. A. Bauer T. Neal R. Merlo A. O. Schmitzer P. R. Hicks G. R. (2007). Chemical genetic identification of glutamine phosphoribosylpyrophosphate amidotransferase as the target for a novel bleaching herbicide in Arabidopsis. Plant Physiol. 144, 1292–1304 10.1104/pp.107.099705 17616508 PMC1914136

[B62] Xuan W. Murphy E. Beeckman T. Audenaert D. De Smet I. (2013). Synthetic molecules: helping to unravel plant signal transduction. J. Chem. Biol. 6, 43–50 10.1007/s12154-013-0091-8 24432124 PMC3606696

[B63] Zhao Y. Dai X. Blackwell H. E. Schreiber S. L. Chory J. (2003). SIR1, an upstream component in auxin signaling identified by chemical genetics. Science 301, 1107–1110 10.1126/science.1084161 12893885

[B64] Ziegler S. Pries V. Hedberg C. Waldmann H. (2013). Target identification for small bioactive molecules: finding the needle in the haystack. Angew. Chem. Int. Edit. 52, 2744–2792 10.1002/anie.201208749 23418026

